# Prevalence, Identification, and Antimicrobial Resistance of Bacterial Pathogens in Farm Animals from Western Kazakhstan

**DOI:** 10.3390/biology15181612

**Published:** 2026-09-12

**Authors:** Laura Dushayeva, Askar Nametov, Aiman Ichshanova, Rashid Karmaliyev, Raushan Rychshanova, Alexandr Shevtsov, Kenzhebek Murzabayev, Dosmukan Gabdullin, Adilbay Karagulov, Balaussa Yertleuova, Ainur Tolegen

**Affiliations:** 1 Veterinary and Agro Technology Institute, Noncommercial Joint Stock Company “Zhangir Khan West Kazakhstan Agrarian-Technical University”, 51 Zhangir Khan St., Uralsk 090009, Kazakhstan; uralsk-laura@mail.ru (L.D.); anametov@mail.ru (A.N.); tore_ias_86@mail.ru (A.I.);; 2 Research Institute of Innovative Technologies, Noncommercial Joint Stock Company “Akhmet Baitursynuly Kostanay Regional University”, 47 A. Baitursynov St., Building 1, Kostanay 110000, Kazakhstan; 3 Laboratory of Applied Genetics, National Center for Biotechnology LLP, 13/5 Korgalzhyn Avenue, Astana 010000, Kazakhstan

**Keywords:** *Escherichia coli*, antimicrobial resistance, food-producing animals, livestock, bacterial monitoring, antimicrobial susceptibility, qPCR, West Kazakhstan Region

## Abstract

Data on the prevalence of bacteria and antimicrobial resistance (AMR) among food-producing animals in Kazakhstan remain limited in scope. As part of this regional cross-sectional descriptive study, specific bacterial taxa and AMR were characterized in farm animals in the West Kazakhstan Region. Between September 2025 and July 2026, samples were collected from 974 individual animals at 48 farms located in 12 administrative districts. Targeted bacteriological isolation was performed for *Escherichia coli*, presumptive *Shigella* spp., *Salmonella* spp., *Yersinia enterocolitica*, *Campylobacter* spp., and *Staphylococcus aureus*, followed by specific real-time PCR. Antimicrobial susceptibility was assessed in 214 *E. coli* isolates using the EUCAST disk diffusion method, and five selected isolates underwent exploratory screening by quantitative PCR (qPCR) for the presence of determinants associated with antimicrobial resistance. A total of 311 bacterial isolates were identified. The predominant taxon was *E. coli*, detected in 22.0% of the animals from which samples were collected and accounting for 68.8% of the isolates; followed by *S. aureus* (3.2% of animals), presumptive *Shigella* spp. (2.9%), *Salmonella* spp. (1.5%), *Campylobacter* spp. (1.5%), and *Y. enterocolitica* (0.8%). Corresponding molecular targets were detected in 303 of 311 presumptively identified isolates (97.4%). No clear age-related gradient in the prevalence of *E. coli* was observed, and this taxon was detected in all three study areas. Phenotypic resistance to at least one antimicrobial agent with an applicable clinical breakpoint according to EUCAST was detected in 2 of 214 *E. coli* isolates (0.9%), including resistance to ceftriaxone or amoxicillin with clavulanic acid. Molecular screening of five selected isolates revealed various determinants associated with antimicrobial resistance and mobile genetic elements, with blaCMY, *mphA*, *qepA_1_2*, *catA1*, and *intI1F165_clinical* being the most frequently detected. These results establish a regional baseline for comprehensive monitoring of bacteria associated with animal husbandry and antimicrobial resistance in Western Kazakhstan.

## Simple Summary

Bacterial infections in farm animals can affect both animal health and human health, as some bacteria can be transmitted from animals to people. This study investigated which bacteria are present in cattle, sheep, goats, and poultry in Western Kazakhstan. We collected nearly 1000 samples and used both traditional laboratory methods and genetic testing to identify the bacteria. The most common bacterium found was *Escherichia coli*, which was present in almost 70% of positive samples. Other bacteria, such as *Staphylococcus aureus* and *Shigella*, were also found, but much less often. Young animals were more likely to carry these bacteria than adults. We also tested whether the bacteria were resistant to antibiotics. Only a small number (about 2%) showed resistance, but genetic testing revealed that some carried hidden resistance genes. These results help us understand the local situation and support the need for ongoing monitoring to protect animal and human health.

## 1. Introduction

Animals used for food production can carry a wide range of bacteria of veterinary and zoonotic significance, including members of the order Enterobacterales and the families Campylobacteraceae and Staphylococcaceae. Some of these microorganisms can be transmitted between animals and humans through direct contact, contaminated environments on farms, water, or animal-derived products, underscoring the close interconnection between animal health, food safety, and public health [1,2,3]. Bacteria of particular veterinary and zoonotic importance include *Escherichia coli*, *Salmonella* spp., *Yersinia enterocolitica*, *Campylobacter jejuni*, and *Staphylococcus aureus* [3,4,5,6,7,8]. However, their epidemiological significance varies considerably. Some species, particularly *E. coli*, include both commensal and pathogenic strains, whereas others are widely recognized as foodborne or opportunistic pathogens. Consequently, the detection of these bacteria in farm animals should be interpreted with consideration of the host species, sample type, clinical context, and characteristics of the isolated strains.

*Escherichia coli* is of particular interest in epidemiological studies, as it is a common component of the human and animal gut microbiota and also includes pathogenic strains capable of causing intestinal and extraintestinal infections [3,9]. It is important to note that both commensal and pathogenic *E. coli* populations can acquire, retain, and spread antimicrobial resistance determinants and, therefore, serve as useful indicator organisms for monitoring antimicrobial resistance in food-producing animal populations. Recent studies have highlighted the wide diversity of antimicrobial resistance determinants and clinically significant *E. coli* clonal lineages circulating among animals raised for meat, as well as their potential exchange between animals, humans, and the environment [10]. Resistant *E. coli* populations and their resistance determinants can be found in the interconnected sectors of animal husbandry, the food industry, human health, and the environment, underscoring their importance within the “One Health” surveillance framework [11].

The significance of these bacteria for veterinary medicine and public health has increased even further due to the global spread of antimicrobial resistance (AMR). Exposure to antimicrobial agents can create selective pressure that promotes the survival of resistant bacterial populations, while horizontal gene transfer contributes to the preservation and spread of resistance determinants among bacterial hosts. Of particular concern among members of the order Enterobacterales are extended-spectrum β-lactamases, AmpC-β-lactamases, plasmid-mediated quinolone resistance determinants, class 1 integrons, insertional sequences, and other mobile genetic elements involved in the acquisition and spread of AMR [12,13,14,15]. Plasmid-mediated colistin resistance poses an additional challenge in the context of the “One Health” concept, as mcr determinants are detected in *E. coli* isolated from food-producing animals and in food products worldwide. In a recent systematic review and meta-analysis, mcr-1 was identified as the predominant mobile determinant of colistin resistance among colistin-resistant *E. coli* strains associated with the food chain [16]. Thus, monitoring such factors is important not only for animal health but also for food safety, as well as for the “One Health” concept, which recognizes the interconnected spread of resistant bacteria and resistance genes among animals, humans, food systems, and the environment.

Monitoring bacterial populations and antimicrobial resistance requires complementary phenotypic and molecular approaches. Culture-based isolation remains important because it yields viable bacterial isolates for subsequent identification and testing for antimicrobial susceptibility. Targeted PCR can complement presumptive culture-based and biochemical identification by detecting specific molecular markers, whereas molecular assays targeting antimicrobial resistance determinants provide information on the genetic characteristics of individual isolates [17,18,19,20]. It is important to note that phenotypic testing for antimicrobial susceptibility and molecular detection of resistance determinants characterize different biological properties: phenotypic testing measures an isolate’s observable response to an antimicrobial agent under standardized conditions, whereas PCR demonstrates the presence of a specific genetic target. Accordingly, the detection of a gene associated with AMR should not automatically be interpreted as evidence of phenotypically expressed resistance, and molecular and phenotypic approaches should be considered complementary rather than interchangeable.

Information on the prevalence of bacteria and antimicrobial resistance in animals used for food production in Kazakhstan remains relatively limited and geographically uneven. Previous studies have demonstrated the presence of antimicrobial-resistant *E. coli* strains in cattle in Kazakhstan [21]. More recently, independent regional studies characterized the determinants of antimicrobial resistance in poultry-associated *E. coli*, as well as the bacterial composition of biological samples collected from cattle and small ruminants in the West Kazakhstan Region [22,23]. However, these studies examined separate and more limited animal populations. Comprehensive regional data covering multiple species of farm animals and combining bacterial isolation, molecular confirmation using targeted markers, and phenotypic testing for antimicrobial susceptibility remain limited.

Therefore, the aim of this study was to describe the prevalence and taxonomic composition of individual bacterial isolates collected from cattle, sheep, goats, and poultry in the West Kazakhstan Region; to perform molecular confirmation of preliminarily identified isolates using targeted real-time PCR; and to characterize the phenotypic susceptibility to antimicrobial agents in the population of isolated *E. coli* strains, supplemented by molecular screening for determinants associated with antimicrobial resistance (AMR) in individual isolates.

## 2. Materials and Methods

**Figure 1:**
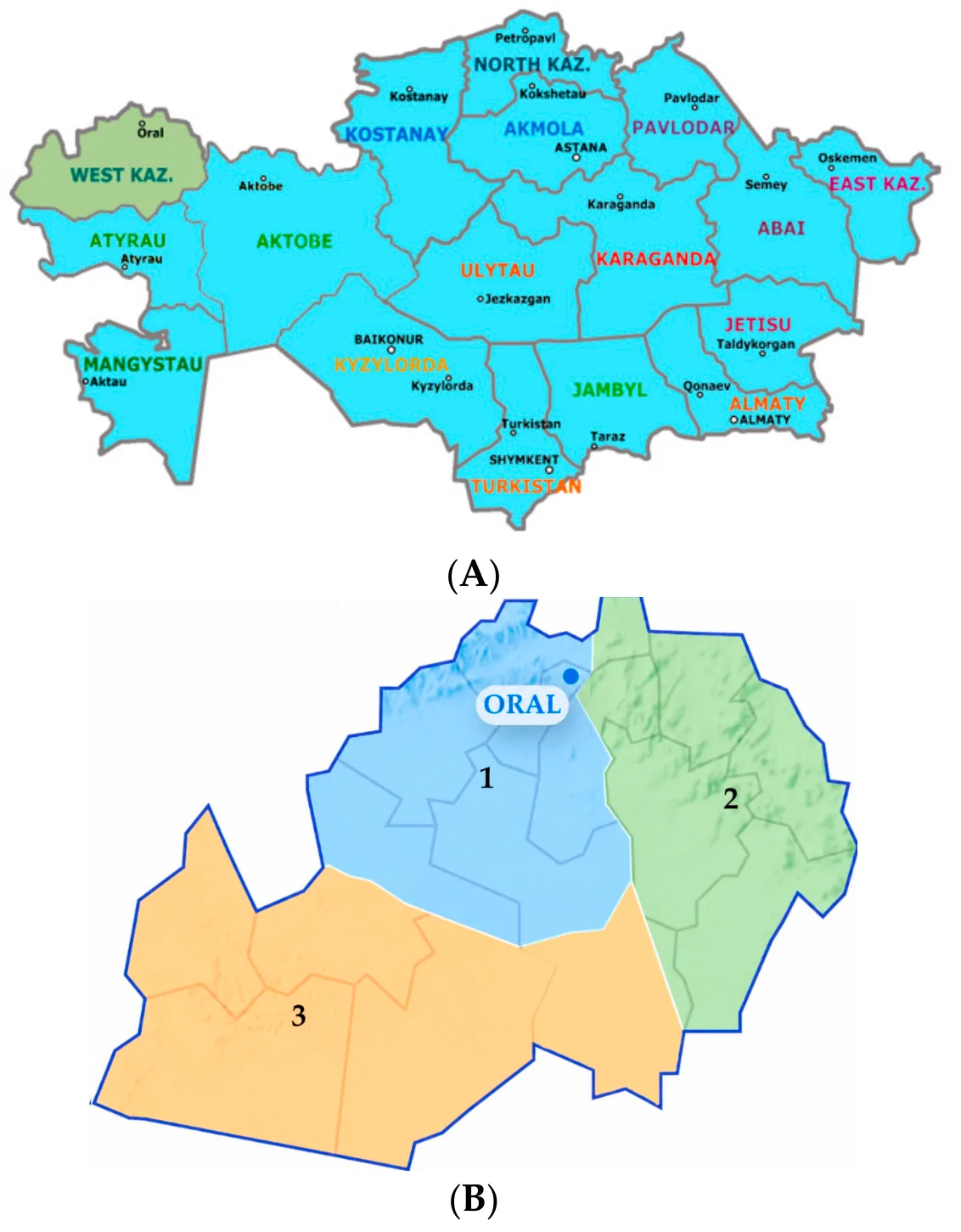
Geographic location of the study area: The Republic of Kazakhstan (**A**) and the West Kazakhstan Region (**B**). The numbered zones indicate the main environmental and climatic regions: (1) Northern and Central districts; (2) Eastern districts; and (3) Southwestern districts. The map was created by the authors using Adobe Photoshop (latest version). No external map source or mapping software was used.

A regional cross-sectional descriptive study was conducted in the West Kazakhstan Region (Kazakhstan) from September 2025 to July 2026. The study covered 48 livestock farms located in 12 administrative districts of the region, with four farms included from each district. Farms were included in the study based on availability and owner consent. For geographic description and subsequent regional comparisons, the surveyed districts were grouped into three study areas—North-Central, Eastern, and Southwestern (Figure 1).

The study population included cattle, sheep, and goats, as well as poultry (chickens, geese, and ducks) of various ages and both sexes. On the participating farms, animals were selected with the owners’ assistance so that the sample included both animals that, according to the owners, exhibited signs of disease and those that appeared healthy. There were no standardized individual clinical records; therefore, clinical status was not used as an analytical variable.

A total of 974 individual animals were sampled, with one biological sample collected from each animal; thus, the 974 biological samples represented 974 different animals. The study material included fecal samples, anal and vaginal swabs, intestinal contents, and oral and nasal swabs, as well as cloacal swabs from poultry. The animals were divided into three age groups: <1 year, 1–3 years, and >3 years (Table 1).

Samples were collected using aseptic techniques with sterile swabs or sterile collection containers, transported to the laboratory in a refrigerated state at a temperature of approximately 4 °C, and processed within 24 h of collection.

According to information provided by the animal owners, antimicrobial agents were administered on some of the farms participating in the study prior to sampling. However, sufficiently reliable information on the specific antimicrobial compounds administered, doses, duration of treatment, and the interval between administration of antimicrobial agents and sampling was not always available. Therefore, prior exposure to antimicrobial agents was not included as an analytical variable.

### 2.1. Bacterial Isolation and Preliminary Identification

Biological samples were processed for the targeted isolation of *Escherichia coli*, presumptive *Shigella* spp., *Salmonella* spp., *Yersinia enterocolitica*, *Campylobacter* spp., and *Staphylococcus aureus*.

For *E. coli*, samples were preliminarily enriched in GRM broth ([Federal State Budgetary Institution “State Scientific Center for Applied Microbiology and Biotechnology.” Moscow Region, Serpukhov City, Obolensk Settlement. Russia]) at 37 °C for 18–24 h and were inoculated onto Endo agar (JSC “NPO Microgen,” Moscow, Russia) and McConkey agar (Conda Laboratories S.A., Madrid, Spain). Suspected colonies were further differentiated on CHROMagar *E. coli* (CHROMagar, La Plaine Saint-Denis, France). To detect suspected *Shigella* spp., samples were enriched in selenite broth (HiMedia Laboratories Pvt. Ltd., Mumbai, India) at 37 °C for 18–24 h and then inoculated onto Salmonella–Shigella (SS) agar (Federal State Budgetary Institution “State Scientific Center for Applied Microbiology and Biotechnology,” Serpukhov, Obolensk, Moscow Region, Russia) followed by incubation at 37 °C for 18–24 h.

For *Salmonella* spp., samples were pre-enriched in buffered peptone water (Federal State Budgetary Institution “State Scientific Center for Applied Microbiology and Biotechnology.” Moscow Region, Serpukhov Municipal District, Obolensk Settlement. Russia) at 37 °C for 18–24 h, followed by selective enrichment in Rappaport–Vassiliadis broth (Federal State Budgetary Institution “State Scientific Center for Applied Microbiology and Biotechnology.” Moscow Region, Serpukhov District, Obolensk Settlement. Russia) at 42 °C for 24–48 h. The enriched cultures were inoculated onto SS agar and incubated at 37 °C for 18–24 h. For *Y. enterocolitica*, enrichment was performed in peptone-sorbitol-bile (PSB) broth (HiMedia Laboratories Pvt. Ltd., Mumbai, India) at 30 °C for 24–48 h, followed by plating onto cefsulodin–irgazan–novobiocin (CIN) agar (Laboratorios Conda S.A., Madrid, Spain) and incubation at 30 °C for 24–48 h.

For *Campylobacter* spp., samples were enriched in Brucella broth (Federal State Budgetary Institution “State Scientific Center for Applied Microbiology and Biotechnology,” Moscow Region, Serpukhov City, Obolensk Settlement, Russia) at 42 °C for 24–48 h and were inoculated onto modified charcoal agar with cefoperazone and deoxycholate (mCCDA; Laboratorios Conda S.A., Madrid, Spain) and onto CHROMagar Campylobacter medium (CHROMagar, La Plaine Saint-Denis, France). The cultures were incubated at 42 °C for 24–48 h under microaerophilic conditions created in an anaerostat (ANAEROSTAT AE-01, Niki MLT LLC, St. Petersburg, Russia) using gas-generating packets (“Kampilogaz” dry gas-generating packets, Inko LLC, St. Petersburg, Russia).

For *S. aureus*, samples were enriched in saline broth (Dry Broth for the Isolation of Staphylococci (NaCl 85.0 ± 2.0), Federal State Budgetary Institution “State Scientific Center for Applied Microbiology and Biotechnology,” Moscow Region, Serpukhov City, Obolensk Settlement, Russia) at 37 °C for 18–24 h and inoculated onto CHROMagar *Staphylococcus aureus* (CHROMagar, La Plaine Saint-Denis, France), and incubated at 37 °C for 18–24 h. Suspected isolates were evaluated using an oxidase test (Oxidase Activity Test Discs (HiMedia Laboratories Pvt. Ltd., Mumbai, India).

Colonies exhibiting characteristic morphology were subcultured to obtain pure cultures. Preliminary identification was based on colony morphology, Gram staining, and biochemical characteristics, including lactose fermentation, indole and hydrogen sulfide production, catalase and oxidase activity, as well as motility (Table 2). In cases where morphologically distinct colonies corresponding to the target group of bacteria were isolated from the same sample, one representative colony of each morphotype was selected for further characterization. Following preliminary identification, one isolate representing each target taxon, isolated from a separate animal, was selected for further analysis.

### 2.2. DNA Extraction and Molecular Confirmation of Bacterial Isolates

Genomic DNA was isolated from pure bacterial cultures using the QIAamp DNA Mini Kit (QIAGEN, Hilden, Germany) according to the manufacturer’s instructions. DNA concentration and purity were assessed using a NanoDrop OneC spectrophotometer (Thermo Fisher Scientific, Waltham, MA, USA), and DNA preparations were stored at −20 °C until molecular analysis was performed.

The bacterial isolates identified during the preliminary stage were subjected to specific real-time PCR using TaqMan hydrolyzable probes. The assays were designed to detect the uidA gene for *Escherichia coli*, the invA gene for *Salmonella* spp., ipaH for putative *Shigella* spp./enteroinvasive *E. coli* (EIEC), foxA for *Yersinia enterocolitica*, mapA for Campylobacter jejuni, and gltS (target: the FMN-binding domain of GltS) for *Staphylococcus aureus* (Table 3). Reactions were performed in final volumes of 25 μL using the BioMaster UDG-HS-qPCR (2×) kit (Biolabmix, Biolabmix LLC, Novosibirsk, Russia) on a QuantStudio 5 real-time PCR system (Applied Biosystems, Thermo Fisher Scientific, Waltham, MA, USA). The concentrations of primers and probes, as well as the amplification conditions, corresponded to the original protocols specified for each assay in Table 3.

In each cycle, nuclease-free water was used as a matrix-free control. Positive controls consisted of DNA isolated from laboratory control isolates whose taxonomic identity had been previously confirmed by whole-genome sequencing.

### 2.3. Assessment of Escherichia coli Susceptibility to Antimicrobial Agents

The susceptibility of *E. coli* isolates to antimicrobial agents was assessed using the standardized EUCAST disk diffusion method on Mueller–Hinton agar (Federal State Budgetary Institution “State Scientific Center for Applied Microbiology and Biotechnology,” Serpukhov, Obolensk, Moscow Region, Russia). Bacterial suspensions were adjusted to a McFarland scale of 0.5 and evenly spread onto the agar surface. The following antimicrobial discs (“NITsF,” St. Petersburg, Russia) were tested: ampicillin (10 μg), trimethoprim–sulfamethoxazole (1.25/23.75 μg), amoxicillin–clavulanic acid (20/10 μg), cefoxitin (30 μg), ceftriaxone (30 μg), imipenem (10 μg), gentamicin (10 μg), tobramycin (10 μg), ofloxacin (5 μg), ciprofloxacin (5 μg), and norfloxacin (10 μg).

The plates were incubated according to the EUCAST disk diffusion method, and the diameters of the inhibition zones were measured in millimeters. The results were interpreted according to the breakpoint criteria of the European Committee on Antimicrobial Susceptibility Testing (EUCAST), version 16.0 [30]. Isolates were classified as susceptible (S), susceptible with increased exposure (I), or resistant (R), where applicable.

Multidrug resistance (MDR) was defined as acquired resistance to at least one antimicrobial agent from three or more classes of antimicrobials, in accordance with Magiorakos et al. [31,32].

### 2.4. Molecular Screening for Antimicrobial Resistance Determinants in E. coli

Five *E. coli* isolates, selected based on the results of disk diffusion testing, underwent exploratory molecular screening for determinants associated with antimicrobial resistance using SYBR Green-based real-time PCR. The primer set included targets associated with resistance to β-lactams, aminoglycosides, tetracyclines, macrolides, sulfonamides, fluoroquinolones, and phenicols, as well as specific mobile genetic elements and determinants associated with resistance to metals and biocides. Primer sequences and target region designations are provided in Supplementary Table S1.

DNA concentration and purity were assessed using a NanoDrop OneC spectrophotometer (Thermo Fisher Scientific, Waltham, MA, USA), and DNA preparations were normalized so that each reaction contained 5 ng of template DNA [30]. Reactions were performed in final volumes of 25 μL using the BioMaster UDG-HS-qPCR Lo-ROX SYBR (2×) kit (Biolabmix, Novosibirsk, Russia) and consisted of 12.5 μL of SYBR Green master mix, 5.0 μL of primer mixture, 5.0 μL of template DNA, and 2.5 μL of nuclease-free water. Amplification and fluorescence detection were performed using the QuantStudio 5 real-time PCR system (Applied Biosystems, Thermo Fisher Scientific, Waltham, MA, USA).

The amplification protocol consisted of an initial denaturation at 95 °C for 3 min, followed by 30 cycles: 95 °C for 15 s, annealing at 62 °C for 20 s, and extension at 72 °C for 10 s; if necessary, the annealing temperature was adjusted to 58–62 °C for individual primer sets. Nuclease-free water was used as a template-free control. Product specificity was assessed by analyzing the melting curve in the range of 65 to 95 °C in 0.5 °C increments.

Amplification signals were considered positive when Ct ≤ 28 [30]. Ct values were not interpreted independently; a reaction was considered positive only if amplification was accompanied by a single specific melting peak. Reactions exhibiting multiple melting peaks or profiles characteristic of primer dimer formation were considered nonspecific and were excluded from further analysis.

### 2.5. Data Analysis

The data were summarized descriptively as absolute numbers (*n*) and percentages (%). For each bacterial taxon, the prevalence was calculated as the proportion of examined animals in which the corresponding taxon was detected, using the total number of animals examined within the corresponding host, age, or geographic group as the denominator. The relative distribution of bacterial taxa among the isolated strains was calculated using the total number of isolates in the respective group as the denominator. The PCR confirmation rate was calculated as the proportion of provisionally identified isolates in which the corresponding molecular target was detected. Differences related to age and region were assessed descriptively; inferential statistical tests were not applied.

## 3. Results

### 3.1. Isolation and Characterization of Bacteria

Of the 974 animals from which samples were collected, a total of 311 bacterial isolates representing six target taxa were identified (Table 4). *Escherichia coli* was the most frequently isolated taxon—214 isolates, corresponding to 22.0% of the animals sampled and 68.8% of all isolates. *Staphylococcus aureus* and, presumably, *Shigella* spp. were isolated from 31 (3.2%) and 28 (2.9%) animals, respectively. *Salmonella* spp. and *Campylobacter* spp. were isolated from 15 animals (1.5%) of each species, while *Yersinia enterocolitica* was isolated from 8 animals (0.8%).

The suspected isolates exhibited colony morphology, Gram stain characteristics, and biochemical profiles consistent with the respective target groups. Subsequently, molecular detection of the target microorganisms was assessed using target-specific real-time PCR.

Among the host groups, *E. coli* was the most frequently isolated taxon: it was detected in 120 of 488 head of cattle (24.6%), in 74 of 357 sheep and goats (20.7%), and in 20 of 129 head of poultry (15.5%) (Table 3). *S. aureus* was isolated from 12 head of cattle (2.5%), 12 sheep and goats (3.4%), and 7 head of poultry (5.4%). *Salmonella* spp. and *Y. enterocolitica* were isolated only from cattle and small ruminants, whereas *Campylobacter* spp. were detected in all three host groups.

Using targeted real-time PCR, the corresponding molecular markers were detected in 303 of 311 presumptively identified isolates (97.4%) (Table 5). Confirmation rates were 97.7% (209/214) for *E. coli*, 96.4% (27/28) for presumptive *Shigella* spp./EIEC, 93.3% (14/15) for *Salmonella* spp., 100% (8/8) for *Y. enterocolitica*, 96.8% (30/31) for *S. aureus*, and the *C. jejuni*-specific mapA target was detected in all 15 presumptively identified Campylobacter isolates (100%).

### 3.2. Distribution of Bacterial Isolates by Animal Age and Study Region

The distribution by age group varied depending on the host group; however, no clear age-related pattern was identified among the bacterial taxa studied (Table 6). In cattle, *E. coli* was detected in 58 of 219 animals under 1 year of age (26.5%), in 40 of 213 animals aged 1–3 years (18.8%), and in 22 of 56 animals over 3 years of age (39.3%). Among sheep and goats, the corresponding proportions were 43 out of 217 (19.8%) and 31 out of 140 (22.1%) for animals under 1 year of age and 1 to 3 years of age, respectively. The distribution of presumed *Shigella* spp. and *S. aureus* also varied across different age groups without a clear, consistent pattern. All poultry included in the study were younger than 1 year of age; therefore, a comparative analysis by age could not be performed for this group of hosts.

From a geographic perspective, *E. coli* remained the most frequently detected taxon in all three study areas: it was detected in 103 of 426 animals (24.2%) in the North-Central region, in 71 of 338 (21.0%) in the Eastern region, and in 40 of 210 (19.0%) in the Southwestern region (Table 7). *S. aureus* was detected in 3.3%, 4.4%, and 1.0% of the sampled animals in the respective regions, whereas *Shigella* spp. was detected in 2.6%, 2.7%, and 3.8%, respectively. *Salmonella* spp., *Y. enterocolitica*, and *Campylobacter* spp. were detected less frequently in all three study districts.

Two additional isolates, Nos. 749 and 758, produced ofloxacin inhibition zones with diameters of 22 and 23 mm, respectively, which corresponds to the EUCAST category “susceptible, increased exposure” (I). No resistance to ampicillin, trimethoprim–sulfamethoxazole, imipenem, or ciprofloxacin was detected. Inhibition zone diameters for norfloxacin were recorded but were not assigned a clinical susceptibility category, as EUCAST version v16.0 specifies threshold values for norfloxacin against Enterobacterales only in the context of uncomplicated urinary tract infections. No isolate met the predefined criterion for multidrug resistance (MDR).

Five selected *E. coli* isolates (Nos. 539, 705, 721, 749, and 758) were further analyzed by qPCR for the presence of determinants associated with antimicrobial resistance, mobile genetic elements, and specific markers of resistance to metals and biocides. qPCR-positive targets were detected in all five isolates, although the molecular profiles varied significantly among the isolates (Table 8; Supplementary Table S3). The most frequently detected targets were blaCMY, mphA, qepA_1_2, catA1, and intI1F165_clinical, each of which was detected in four of the five isolates (80.0%). IS6/257 was detected in three isolates (60.0%), whereas blaPER, oqxA, qac_new_356old, erm(B), silE, sul2, and dfrA14 were detected in two isolates (40.0%).

Isolate No. 758 exhibited the broadest qPCR profile: 30 positive targets spanning multiple classes of antimicrobial resistance and including mobile genetic elements, as well as markers associated with metals and biocides. Isolates No. 721 and No. 749 each yielded six positive targets. Isolate No. 705, for which no R/I classification was assigned among agents with applicable EUCAST clinical cut-off values, yielded 10 qPCR-positive targets.

## 4. Discussion

This study provides baseline regional data on the prevalence of specific bacterial taxa of veterinary and zoonotic significance among farm animals in the West Kazakhstan Region. The study included 974 animals from 48 farms located in 12 administrative districts. This sampling scheme made it possible to characterize the prevalence of bacteria by major livestock groups, age categories, and study districts. Such regional datasets are important for compiling reference information that can subsequently contribute to the development of broader national surveillance systems and surveillance systems within the framework of the “One Health” concept. Overall, *E. coli* was the dominant taxon isolated, detected in 22.0% of the animals examined and accounting for 68.8% of the isolates.

The prevalence of *E. coli* in this study is consistent with its widespread occurrence among food-producing animals, as well as with its established use as an indicator microorganism in the monitoring of antimicrobial resistance. In all livestock systems, *E. coli* acts both as a common commensal microorganism and as an important reservoir of transmissible resistance determinants [10]. It is important to note that the detection of *E. coli* in this study should not, in and of itself, be interpreted as evidence of intestinal disease, since the isolates were not systematically characterized for diarrheal pathotypes or specific virulence profiles. Nevertheless, the widespread prevalence of this microorganism confirms that *E. coli* serves as a practical indicator for regional monitoring of antimicrobial resistance in livestock.

The results obtained complement previous microbiological observations conducted in the West Kazakhstan Region. In an independent study of 260 samples collected from farm animals, 108 bacterial cultures were isolated, including 89 Gram-negative isolates [23]. In another independent study conducted in this region, *E. coli* was confirmed in 40 out of 100 samples collected from a commercial poultry farm [22]. In the present study, *E. coli* also remained the most frequently isolated taxon in cattle (24.6%), sheep and goats (20.7%), and poultry (15.5%), whereas *S. aureus* showed the highest prevalence in poultry (5.4%). *Campylobacter* spp. were isolated at a low frequency in all three host groups, which is consistent with the broad animal reservoir of thermotolerant Campylobacter [33]. Thus, this study expands upon previous regional observations by covering a significantly larger number of hosts, farms, and administrative regions.

An analysis broken down by age did not reveal a consistent, directional pattern in the distribution of bacteria. Among cattle, *E. coli* was detected in 26.5% of animals younger than 1 year, in 18.8% of those aged 1–3 years, and in 39.3% of those older than 3 years, whereas the corresponding proportions for sheep and goats were 19.8% and 22.1% in the two age groups represented. Interpretation of the results is limited by the uneven representation of age groups, particularly the small number of cattle older than 3 years and the absence of older poultry. Thus, the available data do not support the conclusion that there is a consistent age-related gradient in the prevalence of *E. coli*.

Geographic analysis demonstrated the detection of target bacterial taxa in all three study areas. *E. coli* was detected in 24.2% of animals in the North-Central region, in 21.0% in the Eastern region, and in 19.0% in the Southwestern region. Since farms were included in the study based on availability and owner consent, and the comparisons were descriptive in nature, these differences cannot be explained by stocking density, antimicrobial use, housing practices, or other factors at the farm level. The results indicate a broad regional prevalence rather than a risk specific to particular areas.

The level of antimicrobial resistance in *E. coli* strains associated with livestock farming varies significantly depending on the geographic region, host species, production system, set of antimicrobials tested, and sampling strategy. Global monitoring has revealed marked geographic heterogeneity and persistent data gaps in some parts of Central Asia [34]. High resistance rates have been reported in poultry worldwide [35], whereas significantly lower resistance was observed in some of the cattle populations studied [36]. Data from Kazakhstan are also heterogeneous: AMR-associated determinants were detected in 10 of 40 *E. coli* isolates obtained from a commercial poultry farm in Western Kazakhstan, whereas a separate genomic study described a clinical isolate from cattle with a high degree of resistance [21,22]. These studies differ significantly in terms of sampling methods and analytical protocols and should not be compared numerically with the present regional survey. In this study, resistance to at least one clinically relevant drug was observed in only 2 of 214 *E. coli* isolates (0.9%).

Among the phenotypic results, resistance to β-lactam antibiotics deserves special attention. Isolate No. 539 was resistant to ceftriaxone, whereas isolate No. 721 was resistant to amoxicillin with clavulanic acid and produced a cefoxitin inhibition zone below the EUCAST threshold for AmpC screening. Molecular screening of isolate No. 721 additionally identified the blaCMY gene. CMY-type enzymes are among the most widely described plasmid-mediated AmpC beta-lactamases in *E. coli* associated with farm animals [10]. However, the detection of the blaCMY gene by qPCR does not confirm gene expression, enzyme production, or plasmid localization. Profile No. 721 is consistent with AmpC-associated resistance but does not, on its own, confirm AmpC production.

Molecular screening revealed a significantly greater diversity of determinants associated with antibiotic resistance (AR) than would be expected based solely on phenotypic susceptibility. The mismatch between genotype and phenotype is a well-recognized fact, since the detection of a resistance determinant does not confirm its expression, regulatory context, genomic integrity, or contribution to the measured phenotype [37]. Isolate No. 705 illustrates this discrepancy: it contains several qPCR-positive targets associated with antimicrobial resistance, despite the absence of classification into R or I classes among agents with applicable EUCAST clinical cut-off values. The detection of intI1F165_clinical, IS26, IS6/257, and IS6100 further indicates the presence of determinants associated with mobile elements, although qPCR targeting does not allow for the establishment of their physical association or horizontal transfer [38]. These molecular profiles indicate genetic potential associated with resistance rather than directly predicting susceptibility to antimicrobial agents.

Antimicrobial exposure is an important factor in the development of resistance at the population level; however, its contribution could not be assessed in this study due to the lack of reliable information on specific drugs, doses, duration of treatment, and timing of treatment. Data from food-producing animals indicate that restrictions on the use of antimicrobials are generally associated with a decrease in or maintenance of previous resistance levels, although the effects vary depending on the host and resistance determinants [39]. Consequently, neither the low phenotypic resistance observed in this study nor individual molecular data can be attributed to specific antimicrobial use practices. In regions where data on antimicrobial resistance in farm animals remain limited, standardized regional surveys, nevertheless, provide important information for establishing broader surveillance systems within the framework of the “One Health” concept [34]. Thus, this dataset serves as a regional foundation for continuing comprehensive monitoring of AMR in livestock in Kazakhstan.

A number of limitations should be considered when interpreting the results. First, participating farms were included in the study based on availability and owner consent, and animals were selected with the owners’ assistance; consequently, the sample should not be regarded as a probabilistic representation of the entire livestock population in the West Kazakhstan Region. Second, standardized individual clinical records were lacking, which prevented a reliable comparison between outwardly healthy and clinically affected animals. Some farms reported the use of antimicrobial drugs; however, information on specific drugs, doses, duration of treatment, and timing relative to the sampling date was incomplete and, therefore, could not be included in the analysis. The uneven representation of owner groups and age groups—including the absence of older poultry and the relatively small number of cattle older than 3 years—further limits the possibility of direct comparisons by age. These limitations reduce the generalizability of the results and should be taken into account when interpreting regional differences.

The microbiological study focused on six groups of bacteria and, therefore, does not reflect the complete bacterial community of the animals from which samples were collected. Antimicrobial susceptibility testing was limited to *E. coli*, while molecular screening for determinants associated with antimicrobial resistance (AMR) was performed using qPCR only on five selected isolates. Consequently, the qPCR results cannot be extrapolated to the entire collection of 214 *E. coli* isolates, and targeted qPCR does not allow for the determination of gene expression, genomic location, or physical association among the detected determinants. Therefore, the molecular results should be interpreted as characteristics of the selected isolates rather than as a regional resistome.

## 5. Conclusions

This study provides initial descriptive data on the prevalence and distribution of individual bacterial taxa among farm animals in the West Kazakhstan Region. *E. coli* was the most frequently isolated taxon, found in 22.0% of the examined animals and accounting for 68.8% of the isolated strains; however, no clear age gradient was detected in the examined population. Target groups of bacteria were identified in all three study districts, indicating a wide regional prevalence rather than a concentration within a single geographic zone.

Phenotypic antimicrobial resistance among *E. coli* was rarely observed according to the applicable EUCAST clinical breakpoints: two of 214 isolates (0.9%) were classified as resistant to at least one antimicrobial agent. Molecular screening of five selected isolates revealed a variety of determinants associated with antimicrobial resistance and mobile genetic elements, although these profiles did not always correspond to phenotypic susceptibility and cannot be extrapolated to the entire *E. coli* collection. Taken together, these results demonstrate the value of integrating culture-based identification, standardized susceptibility testing, and molecular characterization within a regional surveillance system for AMR. These data establish a regional baseline for future integrated surveillance of livestock-associated bacteria and antimicrobial resistance in Western Kazakhstan.

## Figures and Tables

**Table 1:** Distribution of the examined animals.

№	Animal Species	Number of Biological Samples (*n*)	Age Groups			Study Region
			<1 Year, *n*	1–3 Years, *n*	>3 Years, *n*	
1	Cattle	488	219	213	56	North-Central, Eastern, Southwestern
2	Sheep and goats	357	217	140	0	North-Central, Eastern, Southwestern
3	Chickens, geese, and ducks	129	129	0	0	North-Central, Eastern
	Total	974	565	353	56	

**Table 2:** Key phenotypic characteristics used for the preliminary identification of bacterial isolates.

Target Taxon	Gram Staining and Cell Morphology	Key Phenotypic Characteristics
*Escherichia coli*	Gram-negative rods	Lactose fermentation positive; indole-positive; H_2_S-negative; catalase-positive; oxidase-negative; motile
Presumably *Shigella* spp.	Gram-negative rods	Do not ferment lactose; do not produce H_2_S; catalase-positive; oxidase-negative; nonmotile
*Salmonella* spp.	Gram-negative rods	Lactose fermentation negative; H_2_S positive; catalase positive; oxidase negative; motile
*Yersinia enterocolitica*	Gram-negative coccobacilli	Weak/variable lactose fermentation; H_2_S negative; catalase positive; oxidase negative; motility observed at lower cultivation temperatures
*Campylobacter* spp.	Curved Gram-negative rods	Oxidase-positive; catalase-positive; motile; grow at 42 °C under microaerophilic conditions
*Staphylococcus aureus*	Gram-positive cocci	Catalase-positive; coagulase-positive; characteristic colony morphology on CHROMagar *Staphylococcus aureus*

**Table 3:** Primers and probes used to identify target molecules by real-time PCR.

No.	Bacterium	Gene	Oligonucleotide	Source
1	*E. coli*	uidA_Ecoli_F	GTGTGATATCTACCCGCTTCGC	Frahm, E., Obst, W. (2003) [24]
		uidA_Ecoli_R	AGAACGGTTTGTGGTTAATCAGGA	
		uidA_Ecoli_Probe	6FAM-TCGGCATCCGGTCAGTGGCAGT–BHQ1	
2	*Salmonella* spp.	invA_Salm_F	AGCGTACTGGAAAGGGAAAG	Kasturi, K. N., Drgon, T. (2017) [25]
		invA_Salm_R	ATACCGCCAATAAAGTTCACAAAG	
		invA_Salm_Probe	6FAM-CGTCACCTTTGATAAACTTCATCGCA–BHQ1	
3	*Shigella* spp.	ipaH_F	ACCATGCTCGCAGAGAAACT	Lin WS, Cheng CM, Van KT. (2010) [26]
		ipaH_R	TACGCTTCAGTACAGCATGC	
		ipaH_Probe	CAL Fluor Red 610-TGGCGTGTCGGGAGTGACAGC-BHQ2	
4	*Y. enterocolitica*	foxA_Yenter_F	ACGGCGGTGATGTGAACAA	Wang C. et al. (2014) [27]
		foxA_Yenter_R	GGGTCCACTTGCAGCACATT	
		foxA_Yenter_Probe	FAM-ACCTTCCTTGATGGGCTGCGCTTACTC-BHQ1	
5	*Campylobacter jejuni*	mapA_Cjejuni_F	CTGGTGGTTTTGAAGCAAAGATT	Best, E. L. et al. (2003) [28]
		*mapA_Cjejuni_R*	CAATACCAGTGTCTAAAGTGCGTTTAT	
		mapA_Cjejuni_P robe	FAM-TTGAATTCCAACATCGCTAATGTATAAAAGCCCTTT–BHQ1	
6	*St. aureus*	Saureus_F	TTCTTCACGACTAAATAAACGCTCA	Sun, S. et al. (2025) [29]
		Saureus_R	GGTACTACTAAAGATTATCAAGACGGCT	
		Saureus_Probe	6FAM-CAGAACACAATGTTTCCGATGCAACGT–BHQ1	

**Table 4:** Distribution of bacterial taxa by host group.

Host Group	Number of Animals Sampled, *n*	*E. coli*, *n* (%)	Presumed *Shigella* spp., *n* (%)	*Salmonella* spp., *n* (%)	*Y. Enterocolitica*, *n* (%)	*S. aureus*, *n* (%)	*Campylobacter* spp., *n* (%)	Total Number of Isolates, *n*
Cattle	488	120 (24.6)	15 (3.1)	8 (1.6)	5 (1.0)	12 (2.5)	7 (1.4)	167
Sheep and goats	357	74 (20.7)	10 (2.8)	7 (2.0)	3 (0.8)	12 (3.4)	7 (2.0)	113
Poultry	129	20 (15.5)	3 (2.3)	0	0	7 (5.4)	1 (0.8)	31
Total	974	214 (22.0)	28 (2.9)	15 (1.5)	8 (0.8)	31 (3.2)	15 (1.5)	311

**Table 5:** Molecular confirmation of tentatively identified bacterial isolates.

Preliminary Identification	Molecular Target	Isolates Tested, *n*	Isolates Testing Positive for the Target, *n*	PCR Confirmation Rate, %
*E. coli*	*uidA*	214	209	97.7
*Shigella* spp./EIEC	*ipaH*	28	27	96.4
*Salmonella* spp.	*invA*	15	14	93.3
*Y. enterocolitica*	*foxA*	8	8	100
*S. aureus*	*gltS*	31	30	96.8
Presumably *Campylobacter* spp.	*mapA*	15	15	100
Total	—	311	303	97.4

**Table 6:** Age distribution of bacterial taxa isolated from the selected animals.

Host Group	Age Group	Number of Animals Sampled, *n*	*E. coli*, *n* (%)	Presumed *Shigella* spp., *n* (%)	*S. aureus*, *n* (%)	Other Bacteria *, *n* (%)	Total Number of Isolates, *n*
Large cattle	<1 year	219	58 (26.5)	9 (4.1)	7 (3.2)	4 (1.8)	78
	1–3 years	213	40 (18.8)	4 (1.9)	3 (1.4)	9 (4.2)	56
	>3 years	56	22 (39.3)	2 (3.6)	2 (3.6)	7 (12.5)	33
Sheep and goats	<1 year	217	43 (19.8)	7 (3.2)	6 (2.8)	5 (2.3)	61
	1–3 years	140	31 (22.1)	3 (2.1)	6 (4.3)	12 (8.6)	52
Poultry	<1 year	129	20 (15.5)	3 (2.3)	7 (5.4)	1 (0.8)	31
Total	—	974	214 (22.0)	28 (2.9)	31 (3.2)	38 (3.9)	311

Note: *—“Other bacteria” included *Salmonella* spp., *Y. enterocolitica*, and *Campylobacter* spp.; percentages were calculated using the number of animals sampled in each host age group as the denominator.

**Table 7:** Distribution of bacterial taxa by study region.

Antimicrobial Agent (Disc Concentration)	No. 539	No. 705	No. 721	No. 749	No. 758
Ampicillin (10 μg)	21 (S)	22 (S)	26 (S)	23 (S)	21 (S)
Trimethoprim–sulfamethoxazole (1.25/23.75 μg)	30 (S)	30 (S)	33 (S)	29 (S)	27 (S)
Amoxicillin–clavulanic acid (20/10 μg)	23 (S)	28 (S)	16 (R)	30 (S)	22 (S)
Cefoxitin, screening test (30 μg)	31 (negative)	38 (negative)	18 (positive)	25 (negative)	20 (negative)
Ceftriaxone (30 μg)	19 (R)	27 (S)	30 (S)	33 (S)	29 (S)
Imipenem (10 μg)	29 (S)	29 (S)	32 (S)	27 (S)	25 (S)
Gentamicin (10 μg)	22 ^†^	22 ^†^	23 ^†^	22 ^†^	21 ^†^
Tobramycin (10 μg)	25 ^†^	24 ^†^	27 ^†^	20 ^†^	21 ^†^
Ofloxacin (5 μg)	29 (S)	35 (S)	35 (S)	22 (I)	23 (I)
Ciprofloxacin (5 μg)	40 (S)	31 (S)	34 (S)	30 (S)	26 (S)
Norfloxacin (10 μg)	32 (NI)	37 (NI)	27 (NI)	26 (NI)	23 (NI)

Note: Inhibition zone diameters (mm) are indicated. S—susceptible; I—susceptible, increased exposure; R—resistant; NI—not interpretable. Cefoxitin was used for AmpC screening (cutoff value—19 mm); norfloxacin test results were not clinically interpretable because the EUCAST cutoff value is limited to uncomplicated urinary tract infections. ^†^ Exceeds the EUCAST resistance cutoff value indicated in parentheses.

**Table 8:** Phenotypic susceptibility and qPCR profiles of individual *Escherichia coli* isolates.

Isolate	Phenotypic Data	Unique qPCR-Positive Targets, *n*	Selected qPCR-Positive Targets
539	Resistance to ceftriaxone	14	*ampC*, *blaTEM*, *blaCMY*, *qepA_1_2*, *mphA*, *floR*, *sul2*, *dfrA14*; int1-a-marko, intI1F165_clinical, IS26, IS6100
705	No R/I result available *	10	*blaCTX-M*, *blaCMY*, *oqxA*, *qepA_1_2*, *mphA*, *erm*(B), *catA1*; IS6/257, intI1F165_clinical, qac_new_356old
721	Resistance to amoxicillin–clavulanic acid; positive screening for cefoxitin	6	*blaCMY*, *blaPER*, *qepA_1_2*, *mphA*, *catA1*; IS6/257
749	I to ofloxacin	6	*blaCMY*, *qepA_1_2*, *mphA*, *catA1*; intI1F165_clinical, IS6/257
758	I to ofloxacin	30	A broad profile that includes targets associated with β-lactams, aminoglycosides, tetracyclines, macrolides, sulfonamides, trimethoprim, and fenicol; intI1F165_clinical and other markers associated with motility, metals, and biocides

* Note: R—resistance; I—susceptibility, increased susceptibility. No R/I classification was assigned among agents with clinically applicable EUCAST breakpoints. Complete qPCR profiles and Ct values are provided in Supplementary Table S2.

## Data Availability

The data presented in this study are available from the corresponding author upon reasonable request.

## Supplementary Materials

The following supporting information can be downloaded at: https://www.mdpi.com/article/10.3390/biology15181612/s1, All primer sequences used for the detection of antimicrobial resistance genes in *Escherichia coli* are provided in Supplementary Table S1. The complete profiles of antimicrobial resistance genes and mobile genetic elements identified in the *Escherichia coli* isolates by the qPCR array are provided in Supplementary Table S2. The complete antimicrobial resistance gene and mobile genetic element profiles detected by the qPCR array in selected *E. coli* isolates, including the corresponding Ct values, are presented in Supplementary Table S3.

## Abbreviations

The following abbreviations are used in this manuscript:

AMR	Antimicrobial resistance
ARGs	Antimicrobial resistance genes
CLSI	Clinical and Laboratory Standards Institute
DNA	Deoxyribonucleic acid
ESBL	Extended-spectrum β-lactamase
EUCAST	European Committee on Antimicrobial Susceptibility Testing
MDR	Multidrug-resistant
MIC	Minimum inhibitory concentration
PCR	Polymerase chain reaction
qPCR	Quantitative real-time polymerase chain reaction
WHO	World Health Organization
One Health	One Health approach
BSL	Biosafety level
