# Supplementary material for: Prevalence, Identification, and Antimicrobial Resistance of Bacterial Pathogens in Farm Animals from Western Kazakhstan
Source: Biology (Basel). 2026 Sep 12;15(18):1612. doi: 10.3390/biology15181612 (PMC13604802; doi:10.3390/biology15181612)
Supplement: Supplementary file 1 [file biology-15-01612-s001.zip › Table S1 Primer sequences for detection of antibiotic resistance genes in Escherichia coli.pdf]

Table S 1 - Primer sequences for detection of antibiotic resistance genes in *Escherichia coli*

Note: All primer sequences were obtained from Stedtfeld et al. (2018) [25]

Part 1 (Rows 1–28)

No.	Gene	Primer number	Forward primer (5′→3′)	Reverse primer (5′→3′)
1	aph(3")-ia	435	TAACAGCGATCGCGTATTTCG	TCCGACTCGTCCAACATCAATA
2	tra-A	1526	AAGTGTTCAGGGTGCTTCTGCGC	GTCATGTACATGATGACCAAAA
3	aadA5	98	ATCACGATCTTGCGATTTTGCT	CTGCGGATGGGCCTAGAAG
4	aadA10	426	ACAGGCACTCAACGTCATCG	CGCGGAGAACTCTGCTTTGA
5	blaCTX-M	162	GCGATAACGTGGCGATGAAT	GTCGAGACGGAACGTTTCGT
6	ampC/blaDHA	112	TGGCCGCAGCAGAAAGA	CCGTTTTATGCACCCAGGAA
7	blaFOXnew	1125	CCTACGGCTATTCGAAGGAAGATAAG	CCGGATTGGCCTGGAAGC
8	erm(B)	804	GAACACTAGGGTTGTTCTTGCA	CTGGAACATCTGTGGTATGGC
9	int1-a-marko	336	CGAAGTCGAGGCATTTCTGTC	GCCTTCCAGAAAACCGAGGA
10	aadA2	97	ACGGCTCCGCAGTGGAT	GGCCACAGTAACCAACAAATCA
11	aadA16	427	ACGGTGGCCTGAAGCC	GAATTGCAGTTCCCGTCTGG

No.	Gene	Primer number	Forward primer (5′→3′)	Reverse primer (5′→3′)
12	imp-marko (blaIMP-1)	324	GGAATAGAGTGGCTTAATTC	GGTTTAACAAAACAACCACC
13	oqxA	1577	GAGTCAACCTACCTCCACTATCA	GCTGCGAGTTATCCAGCAG
14	tetA	180	CTCACCAGCCTGACCTCGAT	CACGTTGTTATAGAAGCCGCATAG
15	aphA1	170	TGAACAAGTCTGGAAAGAAATGCA	CCTATTAATTTCCCCTCGTCAAAAA
16	mobA	1524	GCTTCCCGTAACGAGGTAGT	CCTTGAACGGTATCAGCACG
17	ampC	1505	CTGGCGCATACCTGGATTAC	GCCAGTTCAGCATCTCCCA
18	strB	177	GCTCGGTCGTGAGAACAATCT	CAATTTCGGTCGCCTGGTAGT
19	aadA17	428	TGTACGGCTCCGCAGTG	CACGGAATGATGTCGTCGTG
20	NDM new	362-n2-25-15	GGCCACACCAGTGACAATATCA	CAGGCAGCCACCAAAAGC
21	qnrB46,47,48	1579	CGACGTTCAGTGGTTCAGATCTC	GCCAAGCCGCTCCATGAG
22	dfra14	600	CGGATCATGTCATTGTTTCAGG	ATGTTAGAGGCGAAGTCTTGG

No.	Gene	Primer number	Forward primer (5′→3′)	Reverse primer (5′→3′)
23	IS26	1546	ATGGATGAAACCTACGTGAAGGTC	CGGTACTTAATCTGTCGGTGTTCA
24	mphA	812	TCAGCGGGATGATCGACTG	GAGGGCGTAGAGGGCGTA
25	catA1	130	GGGTGAGTTTCACCAGTTTTGATT	CACCTTGTCGCCTTGCGTATA
26	blaSHV-11	1110	TTGACCGCTGGGAAACGG	TCCGGTCTTATCGGCGATAAAC
27	qepA_1_2	1201	GGGCATCGCGCTGTTC	GCGCATCGGTGAAGCC
28	dfrA1	58	GGAATGGCCCTGATATTCCA	AGTCTTGCGTCCAACCAACAG

Part 2 (Rows 29–55)

No.	Gene	Primer number	Forward primer (5′→3′)	Reverse primer (5′→3′)
29	*erm(F)*	23	CAGCTTTGGTTGAACATTTACGAA	AAATTCCTAAAATCACAACCGACAA
30	IS6/257	1560	ATATCGTGCCATTGATGCAGAG	ACCATTGCTACCTTCGTTGAAG
31	*ArmA*	405	TCTTCGACGAATGAAAGAGTCG	GCTAATGGATTGAAGCCACAACC

No.	Gene	Primer number	Forward primer (5′→3′)	Reverse primer (5′→3′)
32	*floR*	913	AACCCGCCCTCTGGATCA	GCCGTCGAGAAGAAGACGAA
33	*blaOXA10*	1506	CGACCGAGTATGTACCTGCTTC	TCAAGTCCAATACGACGAGCTA
34	*dfrA12*	59	CCTCTACCGAACCGTCACACA	GCGACAGCGTTGAAACAACTAC
35	*aadE*	174	TACCTTATTGCCCTTGGAAGAGTTA	GGAACTATGTCCCTTTTAATTCTACAATCT
36	*IS6100*	1561	CGCACCGGCTTGATCAGTA	CTGCCACGCTCAATACCGA
37	*tetX*	196	AAATTTGTTACCGACACGGAAGTT	CATAGCTGAAAAAATCCAGGACAGTT
38	*cmlA1*	127	TAGGAAGCATCGGAACGTTGAT	CAGACCGAGCACGACTGTTG
39	*blaTEM*	1512	CGCCGCATACACTATTCTCAG	GCTTCATTCAGCTCCGGTTC
40	*pcoA*	1303	TGGCGTATGGAGTTTCAATGC	GAATAATGCCGTGCCAGTGAA
41	*sul1 NEW*	363	GCCGATGAGATCAGACGTATTG	CGCATAGCGCTGGGTTTC
42	*intI1F165_clini*	359	CGAACGAGTGGCGGAGGGTG	TACCCGAGAGCTTGGCACCCA

No.	Gene	Primer number	Forward primer (5′→3′)	Reverse primer (5′→3′)
	cal			
43	*aacA43*	423	CTTGGCCTACATTAGATTCAGCTC	GCTCTCAATCTTTGATAGGAGCAG
44	*strA*	176	CCGGTGGCATTTGAGAAAAA	GTGGCTCAACCTGCGAAAAG
45	blaACC-1	28	CACACAGCTGATGGCTTATCTAAAA	AATAAACGCGATGGGTTCCA
46	*tetB*	181	AGTGCGCTTTGGATGCTGTA	AGCCCCAGTAGCTCCTGTGA
47	*silE*	1304	GGTGGAAAGTCATCAGAGGATGA	CAAAGCCCAGCAAGGATGC
48	*sul2*	133	TCATCTGCCAAACTCGTCGTTA	GTCAAAGAACGCCGCAATGT
49	*tetO*	192	CAACATTAACGGAAAGTTTATTGTATACCA	TTGACGCTCCAAATTCATTGTATC
50	*blaPER*	1517	GCAAATGAAGCGCAGATGC	GACCACAGTACCAGCTGGTA
51	*aadB*	429	CCTGCTTGGTGGGCAGAC	CGGCACGCAAGACCTCAA
52	*blaCMY*	108	AAAGCCTCATGGGTGCATAAA	ATAGCTTTTGTTTGCCAGCATCA

No.	Gene	Primer number	Forward primer (5′→3′)	Reverse primer (5′→3′)
53	*KPC*	1523	GCCGCCAATTTGTTGCTGAA	GCCGGTCGTGTTTCCCTTT
54	mcr-1	704	CACATCGACGGCGTATTCTG	CAACGAGCATACCGACATCG
55	*traN*	1525	GCTTGGCGGTCAGCAATT	TTAGGAATAACAATCGCTACACCTTTA
56	*qacdelta_159old*	159	CCCCTTCCGCCGTTGT	CGACCAGACTGCATAAGCAACA
57	*qac_new_356old*	356	GGAGATTTAGCTCATGTAGCTGAAGAA	AAGCTGTTTTATCCCCGTAGCTTTA
