# Supplementary material for: Prevalence, Identification, and Antimicrobial Resistance of Bacterial Pathogens in Farm Animals from Western Kazakhstan
Source: Biology (Basel). 2026 Sep 12;15(18):1612. doi: 10.3390/biology15181612 (PMC13604802; doi:10.3390/biology15181612)
Supplement: Supplementary file 1 [file biology-15-01612-s001.zip › Table S2 Complete antimicrobial resistance gene and mobile genetic element profiles of E. coli isolates detected by qPCR array.pdf]

Supplementary Table S 2

Complete antimicrobial resistance gene and mobile genetic element profiles of *E. coli* isolates detected by qPCR array.

**758(*E. coli*)**		**749(*E. coli*)**		**721 (*E. coli*)**		**705(*E. coli*)**		**539 (*E.coli*).**	
Gene	Ct value	Gene	Ct value	Gene	Ct value	Gene	Ct value	Gene	Ct value
tetX	17.477	mphA	23.462	blaCMY	23,846	blaCTX-M	4,148	int1-a-marko	22,952
tetO	19.735	qepA_1_2	23.604	IS6/257	20,546	oqxA	5,894	ampC	19.183
intI1F165_clinical	21.122	intI1F165_clinical	25.754	qepA_1_2	21,913	catA1	3,721	dfra14	23,419
tra-A	9.275	blaCMY	21.794	mphA	25,15	IS6/257	19,686	IS26	15,7
aadE	9.477	catA1	7.174	catA1	6,562	qac_new_356old	8,1	mphA	18.954
mecA	8.520	IS6/257	7.422	blaPER	4,257	intI1F165_clinical	25,64	qepA_1_2	22.172
dfrA12,	7.153					blaCMY	24,559	floR	13.733
traN	7.727					qepA_1_2	22,85	IS6100	19,588
qac_new_356old	6.855					mphA	23,996	blaTEM	13,778
ArmA	6.163					blaCMY	24.559	pcoA	23,784
oqxA	6.188					erm(B)	7,146	intI1F165_clinical	20,267
mefA	6.000							silE	14,583
blaPER	23.618							sul2	14,399
lnuA	5.324							blaCMY	17,297
sul2	4.134								
sul1	5.246								
silE	4.228								
blaACC-1	5.951								
strA,	5.711								
msr(A)	6.374								
blaOXA10,	4.850								
dfrA1	5.804								
catA1	4.757								
dfra14	5.900								
NDM,	4.386								
erm(B)	8.894								

blaFOX	5.239								
aadA10	5.382								
aadA5	4.392								
aph(3")	4.727								
