# Supplementary material for: Prevalence, Identification, and Antimicrobial Resistance of Bacterial Pathogens in Farm Animals from Western Kazakhstan
Source: Biology (Basel). 2026 Sep 12;15(18):1612. doi: 10.3390/biology15181612 (PMC13604802; doi:10.3390/biology15181612)
Supplement: Supplementary file 1 [file biology-15-01612-s001.zip › Table S3 E. coli isolates detected in the Western Kazakhstan region.pdf]

Table S 3 – E. coli isolates detected in the Western Kazakhstan region

№	Region from which E. coli isolates were collected	Animal species	Isolate number
1	West Kazakhstan Region	Cattle	492
2		Bird	535
3		Bird	539
4		Bird	543
5		Bird	544
6		Cattle	714
7		Cattle	715
8		Cattle	683
9		Cattle	684
10		Cattle	717
11		Small ruminants	733
12		Bird	749
13		Bird	735
14		Cattle	663
15		Cattle	662
16		Cattle	661
17		Cattle	747
18		Cattle	748
19		Bird	750
20		Bird	736
21		Bird	737
22		Bird	738
23		Small ruminants	740
24		Bird	722
25		Cattle	724
26		Cattle	761
27		Cattle	762
28		Bird	766
29		Bird	768
30		Bird	769
31		Cattle	725
32		Cattle	726
33		Cattle	727
34		Cattle	728
35		Small ruminants	758
36		Cattle	759
37		Cattle	760
38		Cattle	763
39		Bird	764
40		Bird	765
41		Small ruminants	755
42		Small ruminants	756
43		Small ruminants	757
44		Cattle	718
45		Bird	721

46		Small ruminants	690
47		Small ruminants	691
48		Small ruminants	692
49		Small ruminants	693
50		Cattle	694
51		Cattle	695
52		Bird	752
53		Bird	751
54		Bird	705
55		Bird	704
56		Small ruminants	701
57		Small ruminants	700
58		Cattle	697
59		Cattle	696
60		Bird	711
61		Bird	710
62		Bird	709
63		Bird	708
64		Bird	707
65		Bird	706
66		Bird	713
67		Bird	712
68		Small ruminants	732
69		Small ruminants	754
70		Small ruminants	730
71		Small ruminants	729
72	Atyrau Region	Small ruminants	666
73		Small ruminants	671
74		Small ruminants	670
75		Small ruminants	672
76		Small ruminants	665
